# Targeting cell cycle arrest in breast cancer by phytochemicals from *Caryto urens* L. fruit ethyl acetate fraction: in silico and in vitro validation

**DOI:** 10.1016/j.jaim.2024.101095

**Published:** 2025-03-12

**Authors:** Ghanshyam Parmar, Jay Mukesh Chudasama, Ashish Shah, Chintan Aundhia, Sunil Kardani

**Affiliations:** Department of Pharmacy, Sumandeep Vidyapeeth Deemed to be University, Piparia, Waghodia, Vadodara, 391760, Gujarat, India

**Keywords:** Breast cancer, *Caryota urens*, Antimetastatic, MCF7 cell line, Bioinformatics, Cell cycle arrest

## Abstract

**Background:**

*Caryota urens*, also known as Shivjata, has been documented in ancient Indian texts for its therapeutic benefits, addressing conditions from seminal weakness to gastric ulcers. This study aims to investigate its contemporary medicinal potential in treating breast cancer.

**Objectives:**

The study focuses on exploring the therapeutic potential of *Caryota urens* fruit against breast cancer, specifically targeting cell cycle genes CDK1, CDC25A, and PLK1 through bioinformatics, network pharmacology, and in vitro validation.

**Materials and methods:**

Using mass spectrometry and nuclear magnetic resonance (NMR), 60 key phytoconstituents from *Caryota urens* fruit were identified. Bioinformatics analysis, integrating Gene Cards and GEO databases, 15,474 breast cancer-associated genes focusing on the HR+/HER2-subtype were identified. Molecular docking and qPCR validated the interactions of key phytoconstituents, particularly Episesamin, with CDK1, CDC25A, and PLK1. In vitro studies were conducted on the MCF7 cell line, supplemented by ROC and survival analyses to evaluate diagnostic and therapeutic potential.

**Results:**

The bioinformatics analysis identified CDK1, CDC25A, and PLK1 as pivotal genes regulating cell cycle progression and breast cancer tumorigenesis. Network pharmacology and in vitro studies indicated that phytoconstituents, especially Episesamin, downregulated these genes in breast cancer cells. Molecular docking and qPCR confirmed these interactions, and ROC and survival analyses underscored their diagnostic and therapeutic significance.

**Conclusions:**

This study suggests that *Caryota urens* fruit extract, particularly Episesamin, may inhibit breast cancer metastasis by downregulating CDK1, CDC25A, and PLK1, offering promising new strategies for targeting the cell cycle in breast cancer and emphasizing the value of integrating bioinformatics with experimental methods in cancer research.

## 1 Introduction

Breast cancer (BC) is a leading cause of cancer-related mortality globally, attributed to its multifactorial etiology and molecular complexity. It primarily arises from unchecked proliferation of ductal or lobular cells in the breast, driven by genetic, hormonal, lifestyle, and environmental factors. Key genetic risk factors include mutations in BRCA1 and BRCA2, while prolonged estrogen exposure contributes hormonally. Environmental exposures, such as radiation, along with lifestyle factors like alcohol consumption and obesity, further elevate risk [1,2]. The pathogenesis of breast cancer involves a series of molecular alterations, facilitating tumour growth, evasion of apoptosis, angiogenesis, and invasion of surrounding tissues [3]. The PI3K/AKT/mTOR and MAPK/ERK signalling pathways are pivotal in driving malignancy. The tumour microenvironment, comprising immune cells and fibroblasts, plays a crucial role in tumour progression. Breast cancer subtypes-primarily invasive ductal carcinoma (IDC), invasive lobular carcinoma (ILC), and ductal carcinoma in situ (DCIS)-are classified based on HER2 and hormone receptor status, influencing prognosis and treatment strategies [[4], [5], [6]].

Research into herbal medicines as alternatives to conventional breast cancer treatments, such as surgery, chemotherapy, and immunotherapy, is gaining attention due to their potential to mitigate adverse side effects. Standard treatments, though effective, can cause both short-term issues (e.g., nausea, hair loss) and long-term complications (e.g., organ damage). This study advocates for using phytochemicals, like those from *Caryota urens (CU)*, for their low toxicity, good efficacy, and cost-effectiveness. *Caryota urens*, from the Arecaceae family, holds promise as a natural resource with therapeutic potential, symbolizing hope for better, less harmful cancer treatments through its botanical properties [7]. *Caryota urens*, highlighted in ancient Indian texts like *Sushruta Samhita* and *Charak Samhita*, is used for treating seminal weakness, urinary issues, and headaches. Its therapeutic applications include treatment of gastric ulcers, snake bites, migraines, and rheumatic swelling. Additionally, it promotes hair growth, exhibits antibacterial and antioxidant properties, and shows potential in cancer treatment [8].

Network pharmacology, introduced by Hopkins in 2007, integrates data science and clinical practice to enhance drug discovery [9]. It examines the synergistic effects and mechanisms of traditional herbal medicines through in-silico “protein-compound/disease-gene” network construction. This study focuses on *Caryota urens* (*C. urens*) to elucidate its molecular mechanisms in treating breast cancer, providing insights into its pharmacological actions. The findings suggest that network pharmacology could accelerate the identification of active compounds for laboratory validation and enhance understanding of *C. urens* as a potential therapeutic agent for breast cancer, offering a promising approach for new drug development [10,11].

Targeting CDK1, CDC25A, and PLK1 offers a promising therapeutic strategy in breast cancer due to their pivotal roles in cell cycle regulation. CDK1, essential for G2 to mitosis transition, is frequently overexpressed in breast cancer, promoting uncontrolled proliferation. Inhibition of CDK1 induces cell cycle arrest. Similarly, CDC25A, which activates CDKs, is overexpressed, contributing to chemoresistance by driving the cell cycle despite DNA damage. Targeting CDC25A may induce apoptosis. PLK1, critical for mitosis, is also overexpressed in aggressive breast cancers, correlating with poor prognosis. Inhibiting these targets could improve therapeutic efficacy, overcome resistance, and enable personalized treatments [[12], [13], [14]].

This study highlights the therapeutic potential of *C. urens* fruit extract against breast cancer, particularly focusing on its phytoconstituent, Episesamin. Leveraging bioinformatics tools and experimental validation, the research explores the interactions between *C. urens* components and key genes, including CDK1, CDC25A, and PLK1, which are crucial for cell cycle regulation and often dysregulated in breast cancer. By utilizing databases like Gene Cards and GEO, the study identifies critical targets within the tumor microenvironment (TME) for therapeutic intervention. Experimental validation with the MCF7 cell line confirms the extract's inhibitory effects on tumour growth and metastasis. Molecular docking studies assess the binding affinity of Episesamin to target genes, while qPCR analyses gene expression changes. This integrative approach provides insights into breast cancer biology and highlights *C. urens* as a promising candidate for developing targeted treatments, especially for the HR+/HER2-subtype, potentially enhancing therapeutic outcomes in challenging cancer cases.

## 2 Material and methods

### 2.1 Data collection

The gene expression profiles GSE226339 was retrieved from the gene expression omnibus (GEO) database (https://www.ncbi.nlm.nih.gov/geo/). The GSE226339 expression profile comprises three Cancer stem cells (CSC) and three control groups, and study was performed by using MCF 7 cell line [15].

### 2.2 Identification of differentially expressed genes (DEGs)

To determine the DEGs, we created a Venn diagram and used the LIMMA method. A statistically significant difference was seen between the two groups when the p-value was less than 0.05 and the Log Fold Change was more than 0.5. A Venn diagram containing frequent genes of breast cancer was generated using the DEGs genes retrieved from the GSE226339 dataset. A gene search was conducted in Gene Cards and the GEO dataset using the phrase “Breast cancer” in GSE226339 datasets. In the screening process, GSE226339 was subjected to the following criteria: a p-value of less than 0.05 and a Log FC value of less than 0.5, then a Venn diagram was created using the aforementioned GSE datasets and gene card BC genes in FunRich 3.1.3 [16,17].

### 2.3 Collection and authentication of plant materials

The dried fruits of *C. urens* was collected during the winter from the Botanical Garden, Maharaja Sayajirao University, Vadodara. The fruits were identified and authenticated by Dr. Padmanabh S Nagar, botanist at Maharaja Sayajirao University, Vadodara and voucher specimen was deposited and stored at Department of Pharmacy, Sumandeep Vidyapeeth. The dried fruits were washed and dried hot air oven at 40 °C for 40–45 min. The dried fruits were grinded to coarse powder using a mixer grinder and further stored in sealed container in refrigerator till further use (Fig. S1).

### 2.4 Preparation of extract

The crude ethanolic extract of CU was prepared using cold maceration method. Briefly, 200 grams of dried powder of sample was macerated with 1 l of ethanol with occasional stirring. At the end of maceration process, the extract was strained through Whatman filter paper and filtrate was concentrated using rotary evaporator (RV10, IKA Germany). The 25.4 % (w/w of dry powder basis) yield of crude ethanolic extract was calculated and stored at 4 °C till further use.

### 2.5 Preparation of flavonoid rich fractions

For flavonoid rich fractionation, the crude ethanolic extract (50 g) was dissolved in distilled water and stirred for 2 h and partitioned with equal volume of chloroform (three times) to remove polyphenols. The aqueous layer was then separated and underwent a second partitioning step with equal volume of ethyl acetate (three times). The resulting ethyl acetate and aqueous fractions (EA fraction) were collected separately. The ethyl acetate fraction obtained from the separation process was further stored in refrigerator at 4 °C till further utilized for in vitro study [18,19].

### 2.6 Screening of chemical constituents of *C. urens*

In order to identify the phytoconstituents of *C. urens*, we looked at published literature as well as mass and NMR data [20]. All chemicals were searched for information on their 3D structures and physiochemical properties in PubChem (https://pubchem.ncbi.nlm.nih.gov/) using the Phytochemical name. Every single canonical SMILE for the active components was obtained from PubChem. Then, utilizing the online platforms TCMSP and SwissTargetPrediction (http://www.swisstargetprediction.ch/), the complete pharmacokinetic characteristics of the active chemical component were computed using the Canonical SMILES. The phytoconstituents and their target genes were retrieved and identified from above online tools and databases [21].

### 2.7 Gene function annotation of DEGs of BC and *C. urens*

The GO analysis categorizes gene functions into biological process (BP), cellular component (CC), and molecular function (MF). The KEGG database analyses pathways, and DAVID (https://david.ncifcrf.gov/) provides comprehensive gene annotation. The common targets were annotated with GO keywords and pathway enrichment was analysed using the “*Homo sapiens*” species in DAVID. To determine which pathways were enriched, a likelihood score lower than 0.05 was utilized. The GO annotation and KEGG pathways were displayed using bubble maps that were made using SRplot (https://bioinformatics.com.cn/srplot) [22,23].

### 2.8 Protein–protein interaction (PPI) network and other network construction

Building a PPI network of the DEGs of BC and *C. urens* was accomplished by utilizing the STRING database (Version 12.0) (http://string-db.org/) [24]. The interaction was deemed significant when the composite score was greater than 0.4. Using Cytoscape 3.10.1 (http://cytoscape.org/), we visualized the interaction network and conducted more in-depth studies to find the hub genes based on their degree of connectivity and of each node. The other networks like compound target network and compound target pathway network were also constructed by using Cytoscape 3.10.1 [25,26].

### 2.9 Molecular docking

Molecular docking facilitates the identification of interactions of ligands to their correlating proteins. For the validation of the results of this study, molecular docking was used. The SDF file formats of the 3D structure of active ingredients were downloaded from the PubChem database and optimized these structures, the PDB format file of hub CDK1, CDC25A, and PLK1 genes were downloaded from Protein Data Bank (RCSB) PDB, (http://www.rcsb.org/). The finest protein crystal structure (smaller resolution value, complete structure, and human protein) was selected for docking. The ligand and protein molecules were charged, hydrogenated, and normalized using AutoDockTool, and PDBQT file format was created. Molecular docking was performed between the refined ingredients and protein using AutoDock Vina. To calculate the binding energy, the Lamarckian genetic algorithm was selected, and Discovery Studio 2021 Client software was used for the visualization of the docked results. By measuring the strong affinity between chemicals and their related targets, this stage aimed to investigate the binding energy between them; the higher the affinity, the lower the binding energy [27,28].

### 2.10 Cell cultures

The MCF-7 cell line was cultured in DMEM medium with 10% fetal bovine serum, penicillin, and streptomycin at 37 °C in a humidified environment with 5% CO_2_. For growth assessment, cells were seeded in 6-well plates at approximately 5 × 10³ cells per well and counted daily for up to 10 days, using trypsin/EDTA for dissociation and a hemacytometer for counting. Each condition was performed in triplicate for reproducibility. For active renin secretion, cells were plated in 24-well plates at 5 × 10⁴ cells per well and allowed to reach confluence. Controls included untreated and vehicle-treated cells. RNA was extracted using a TRIzol-based method, with cDNA synthesized for qPCR analysis using SYBR Green Master Mix and specific primers. Relative gene expression was quantified using the 2^-ΔΔC^^^t^^ method, normalizing to GAPDH, with statistical significance assessed by one-way ANOVA and Tukey's test (p < 0.05) [29].

### 2.11 Isolation of total RNA (trizol method)

RNA isolation followed the manufacturer's protocol using an Invitrogen total RNA separation kit. Cells were disrupted using Trizol, releasing RNA, with chloroform facilitating phase separation: RNA in the aqueous phase, proteins in organic and interphase phases. Upon reaching 80% confluence, cells were treated with 1 mL Trizol on the culture plate to create a homogenized paste. After transferring to Eppendorf tubes, 200 μL of chloroform was added and shaken for 15 s, incubated for 2–3 min at room temperature, then centrifuged at 14,000 rpm and 4 °C for 15 min. The aqueous layer was transferred, mixed with 500 μL isopropanol, and chilled for 10 min to precipitate RNA. The pellet was washed with 1 mL 75% ethanol and centrifuged at 10,000 rpm for 5 min at 4 °C. After drying, the RNA pellet was dissolved in TE buffer [30,31].

### 2.12 Reverse transcriptase PCR (RT-PCR)

Thermo Scientific's Verso cDNA synthesis kit manual was used for cDNA synthesis, and the Invitrogen Thermo Script RT-PCR System instruction was used for amplification. After the amplification, agarose gel electrophoresis was used to sort the PCR product. In real-time PCR research, the primers are used in the following order [32].

### 2.13 Survival analysis therapeutic responder analysis

The prognostic value of CDK1, CDC25A, and PLK1 expression in breast cancer (BRCA) was analysed using the K-M Plotter database (www.kmplot.com), assessing gene expression and survival outcomes, including overall survival (OS) and relapse-free survival (RFS) through Kaplan-Meier plots [33,34]. Transcriptomic data from 3104 BRCA patients were evaluated for biomarker potential in predicting chemotherapy and hormone therapy responses, using ROC plots and Mann-Whitney tests based on pathological response or 5-year relapse-free survival [35]. Results suggest these genes as valuable markers for survival and treatment response in BRCA.

## 3 Results

### 3.1 Identification of differentially expressed genes (DEGs)

**Fig. 1:**
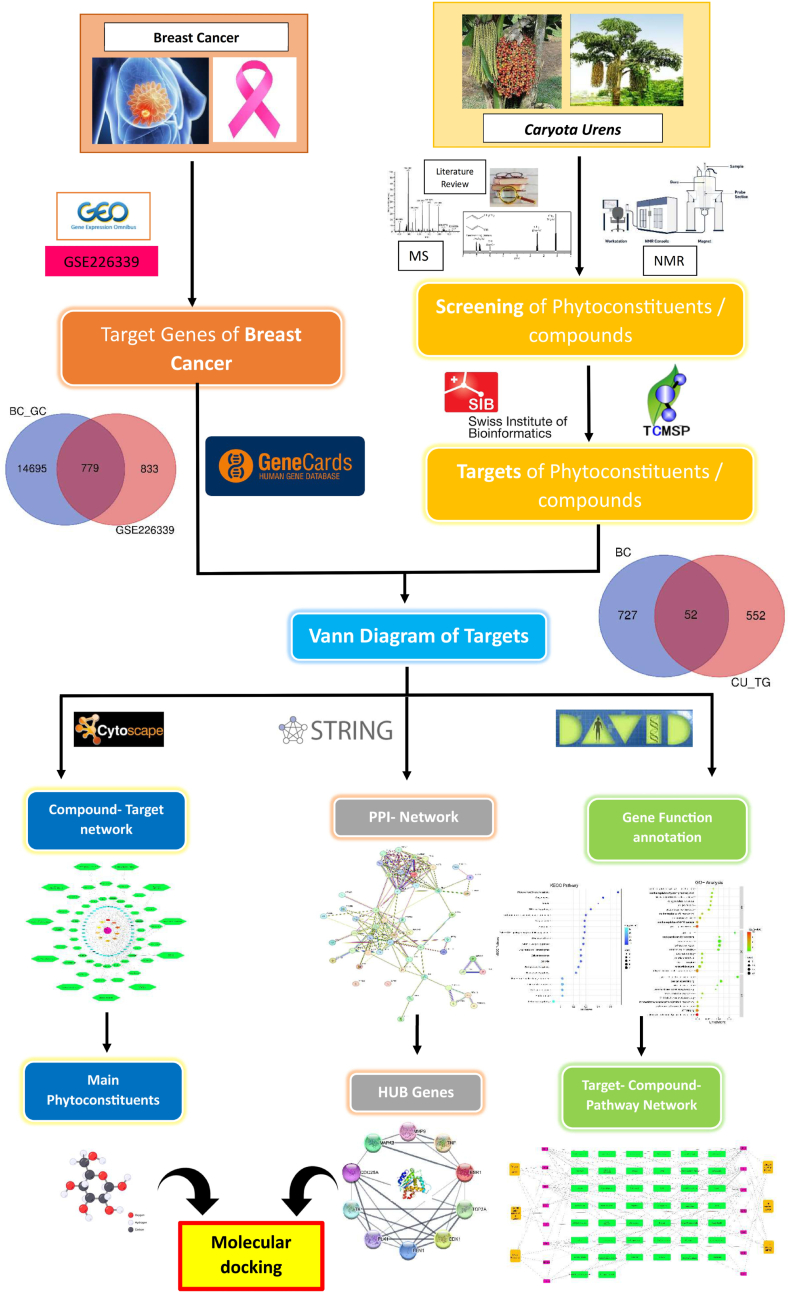
Bioinformatics data from GEO and gene card database on breast cancer and phytoconstituents targets of *C. urens*, differentially expressed genes (DEGs) and their GO, KEGG, and network pharmacology with docking diagram illustrating the entire study technique.

**Fig. 2:**
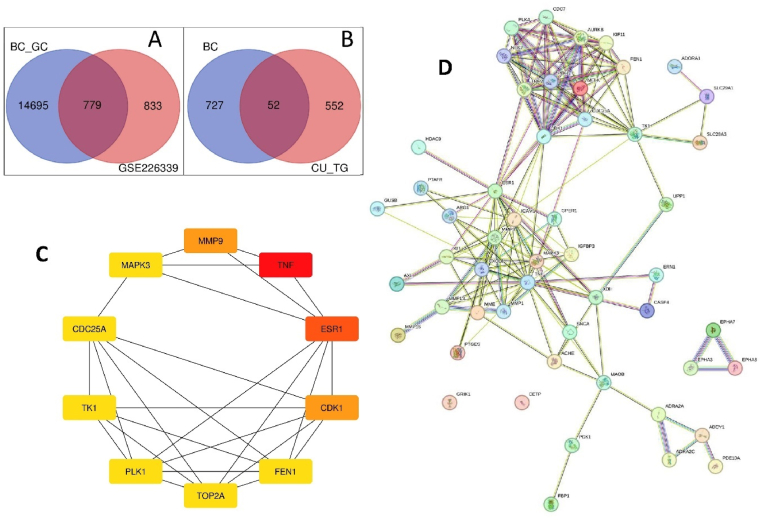
Venn diagrams showing overlapping genes of **(A)** Breast cancer (BC_GC) retrieved from gene card and GEO datasets (GEO-CG) (GSE226339) the common 779 gens DEGs Breast cancer **(B)** DEGs Breast cancer (BC) and *C. urens* target genes the common 52 gens DEGs of BC and *C. urens*. **(C)** Top 10 Hub genes screened based on their degree. **(D)** PPI Network of 52 intersected key targets (*C. urens* and *Breast cancer*).

The research methodology is outlined in Fig. 1, employing the LIMMA package for identifying differentially expressed genes (DEGs) from the GSE226339 dataset, using the Benjamini and Hochberg method for p-value correction. Statistically significant differences were defined as p-value <0.05 and Log Fold Change (Log FC) > 0.5 [15]. A Venn diagram (Fig. 2A) illustrated common genes associated with breast cancer, revealing 779 shared genes from 1662 DEGs identified from GSE226339 against 15,474 genes from Gene Cards. The screening criteria were p-value < 0.05 and Log FC < 0.5. The study focused on potential targets for further analysis involving phytoconstituents and disease target genes, with the final screened genes represented in Fig. 2A. The keywords “breast cancer” were used to refine searches in Gene Cards and the Gene Expression Omnibus (GEO) dataset.

### 3.2 Screening of chemical constituents of *C. urens*

The active phytoconstituents of *Caryota urens* were identified through mass and NMR analysis and confirmed by literature [20], with PubChem used to obtain 3D structures and physicochemical properties. The 92 initial compounds, 60 active components were selected based on pharmacokinetic analysis via TCMSP and SwissTargetPrediction [36]. These were associated with 604 target genes, narrowing to 52 common genes with disease-related genes (779) identified via a Venn diagram (Fig. 2B). After pharmacokinetic screening, the compounds were further refined by criteria such as molecular weight ≤500 Da ≤ 5 rotatable bonds, TPSF between 20 and 130, X-log P between −0.7 and 6, log S ≤ 6, and bioavailability close to 0.55, resulting in 16 promising compounds. Highest docking scores are detailed in Table 1, highlighting the therapeutic potential of *C. urens* for breast cancer [37,38].

**Table 1:** Primers details of selected gene.

Sr. No.	Gene Name	Sequence	Product length (bp)
1	CDK 1	F - TATCCCTTCGCCTGTTCCTC	241
		R - CACACACACACCTCCAATCG	
2	CDC25A	F - AAATGGTTTCTGGCAGCCTG	212
		R - CTCCCCACACAGCAGACTTA	
3	PLK1	F - GTGCCTCACTTTCCTCCTCT	151
		R - GCCGAACACCAACTCACTTT	
4	ACTB	F - AACAGACTCCCCATCCCAAG	202
		R - CCAGAGGCGTACAGGGATAG	

### 3.3 Gene function annotation of DEGs of BC and *C. urens*

DAVID (http://david.ncifcrf.gov/), gene annotation and KEGG pathway analyses were conducted on 52 *Caryota urens* targets for breast cancer treatment. GO annotation identified 138 biological processes (BP), 34 cellular components (CC), and 34 molecular functions (MF), with the top 30 displayed in Figs. S2A–C using a p-value threshold of p < 0.05. KEGG analysis anticipated 32 pathways relevant to breast cancer, with the top 30 shown in Fig. S2D. Visualized through SRplot bubble maps (https://bioinformatics.com.cn/srplot), the GO and KEGG analyses illustrate *C. urens*'s molecular mechanisms, highlighting its potential as a breast cancer therapeutic [23,[39], [40], [41]].

### 3.4 Protein–protein interaction (PPI) network and other network construction

Protein-protein interaction (PPI) analysis (Fig. 2C) conducted using Cytoscape revealed 52 nodes and 158 edges, highlighting key interconnections among targets [42]. Using the CytoHubba plugin, ten hub genes, including TNF, ESR1, CDK1, and MMP9, were identified for their high connectivity, indicating potential therapeutic relevance (Fig. 2D) [43]. A compound-target network (Fig. S3A) mapped interactions between fifteen active compounds of *Caryota urens* and 52 target genes, underscoring their relevance to breast cancer [44]. DAVID analysis identified 20 KEGG-enriched pathways, forming a target-pathway network with 103 nodes and 164 edges (Fig. S3B) [45]. These results emphasize the multi-target, multi-pathway therapeutic potential of *C. urens* in breast cancer treatment [27].

### 3.5 Molecular docking

**Fig. 3:**
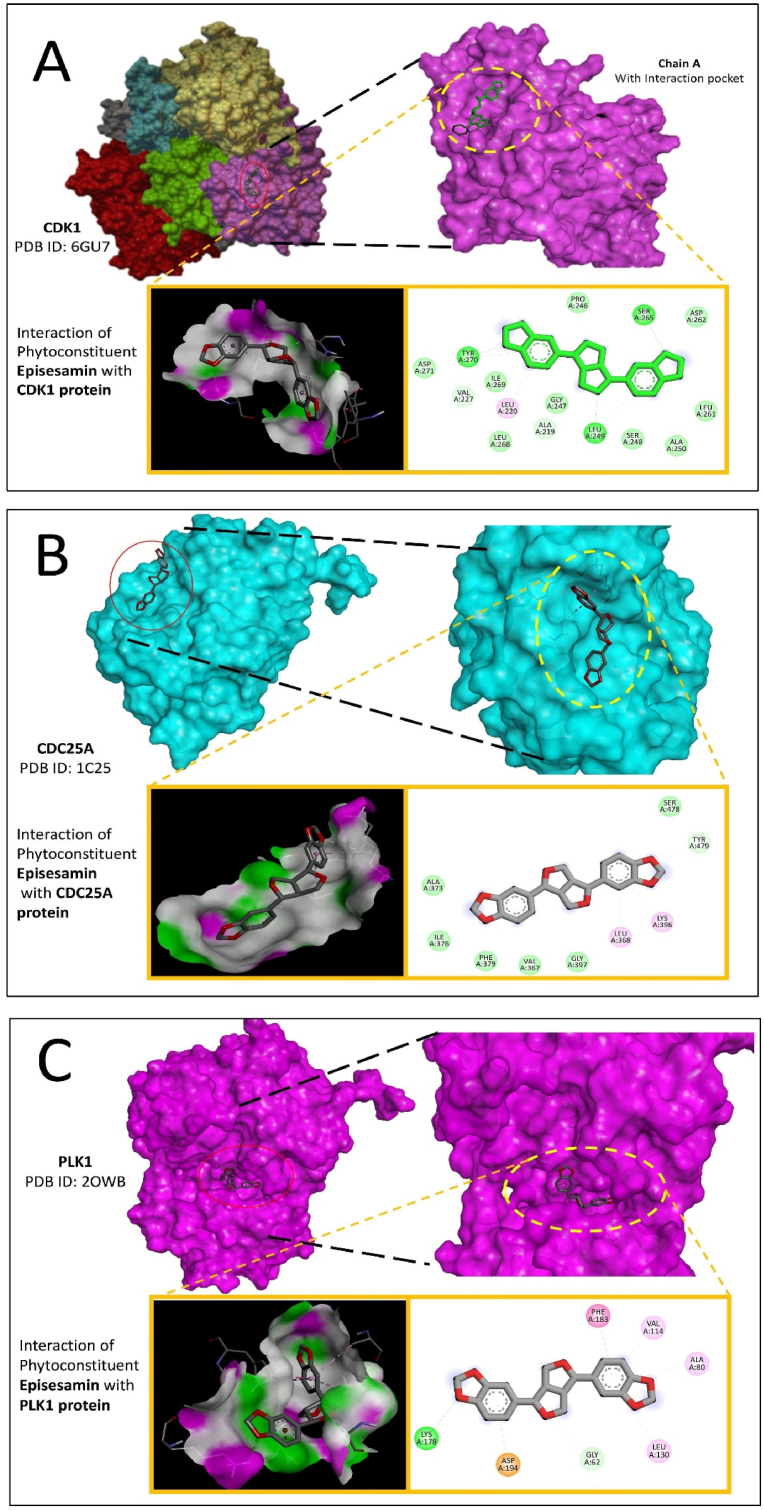
Binding interactions of Episesamin with receptors. (A) CDK1 (PDB ID: 6GU7; score: 8.3), (B) CDC25A (PDB ID: 1C25; score: 7.9), and (D) PLK1 (PDB ID: 2OWB; score: 9).

**Fig. 4:**
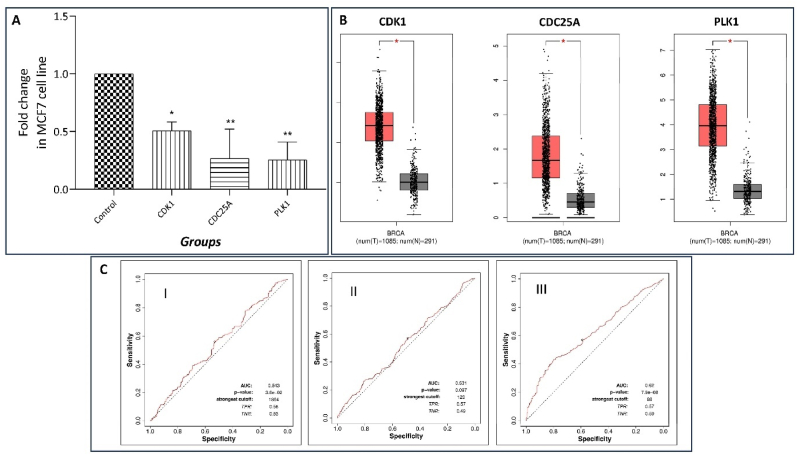
**(A)** The effect of CU on mRNA expression of CDK1, CDC25A, and PLK1 against control on MCF7 cell line. **(B)** CDK1, CDC25A, and PLK1 expression was expressed in box plot based on BRCA data. **(C)** CDK1 (I), CDC25A (II), and PLK1 (III) ROC plot.

Molecular docking of proteins and refined components was performed using AutoDock Vina, employing the Lamarckian genetic algorithm to compute binding energies [46,47]. Results analysed with Discovery Studio 2021 Client software revealed that the active components of *C. urens* exhibited superior binding affinity to breast cancer targets compared to other substances, as shown in Table 2. Notably, CDK1, CDC25A, and PLK1 emerged as critical genes in the cell cycle pathway through docking and KEGG pathway analysis (Fig. 3). Differential expression of these genes was validated using boxplots from the GEPIA database (Fig. 4B), supporting the hypothesis that Epassasamine from *C. urens* may regulate BRCA [48].

**Table 2:** Docking results for interaction with selected phytoconstituents of *C. urens* and top 3 genes of DFU.

Comp ID	Name of Compound	CDK1 (6GU7)	CDC25A (1C25)	PLK1 (2OWB)
COM10	Protocatechuic acid	−5.1	−5.8	−6
COM13	3,4-Dihydroxyphenylaceticacid	−5.3	−5.8	−6.3
COM16	5-(3,4,-dihydroxyphenyl)-Valero lactone	−6.3	−5.9	−7.1
COM18	Dihydrosinapic acid	−5.1	−5.2	−6.5
COM19	3-Hydroxy-3-(3-hydroxyphenyl)propionic acid	−5.3	−5.7	−6.6
COM20	3-Methoxysinensetin	−6.3	−6	−7.7
COM22	3-Methoxynobiletin	−5.9	−5.6	−7.8
COM30	Isorhamnetin	−7.5	−6.5	−8.6
COM44	Episesamin	**−8.3**	**−7.9**	**−9**
COM51	3,4-DHPEA-AC	−5.9	−5.5	−6.6
COM53	p-HPEA-AC	−5.4	−5.4	−6.7
COM54	Mellein	−5.7	−5.8	−7
COM55	Scopoletin	−5.6	−6	−7.3
COM56	3-Methylcatechol	−4.6	−4.6	−5.6
COM60	Thymol	−4.8	−4.7	−6.2
COM60	Pyrogallol	−5	−4.8	−5.2

### 3.6 Effect of CU on mRNA expression levels of CDK1, CDC25A and PLK1 on MCF 7 cell line

In the present study the treatmnet of MCF7 cell treated with the CU (200–500 μL) fraction for 24 h and significantly down regulation of the CDK1, CDC25A and PLK1 which is involved in cell cycle, and responsible for cell cycle arrest in tumour cells of breast cancer. The CDK1, CDC25A and PLK1 was measured as a fold change based on their C_t_ value obtained using qPCR in MCF7 cell line. The relative gene expression of targeted genes is determined with reference to house keeping gene β-actine in triplicate. The CU has shown significant down regulation of the CDK1(P < 0.01), CDC25A (P < 0.01) and PLK1(P < 0.01) on MCF7 cell line [49,44] Fig. 4A.

The multivariate logistics regression analysis was applied to evaluate the performance of the panel of 3 genes (CDK1,CDC25A and PLK1) as biomarkers for breast cancer prediction. The results showed a good performance with a high level of efficiency for breast cancer prediction using different models of these 3 genes. The ROC analysis (Fig. 4C) showed that CDK1 gene (AUC = 0.543 and P-value = 3.6e^−02^), CDC25A gene (AUC = 0.531 and P-value = 0.097) and PLK1 gene (AUC = 0.62 and P-value = 7.9e^−08^). As per ROC plotter (https://rocplot.org/) for breast cancer, with Chemotherapy in pathological complete response (1775) [50].

### 3.7 Survival analysis therapeutic responder analysis

**Fig. 5:**
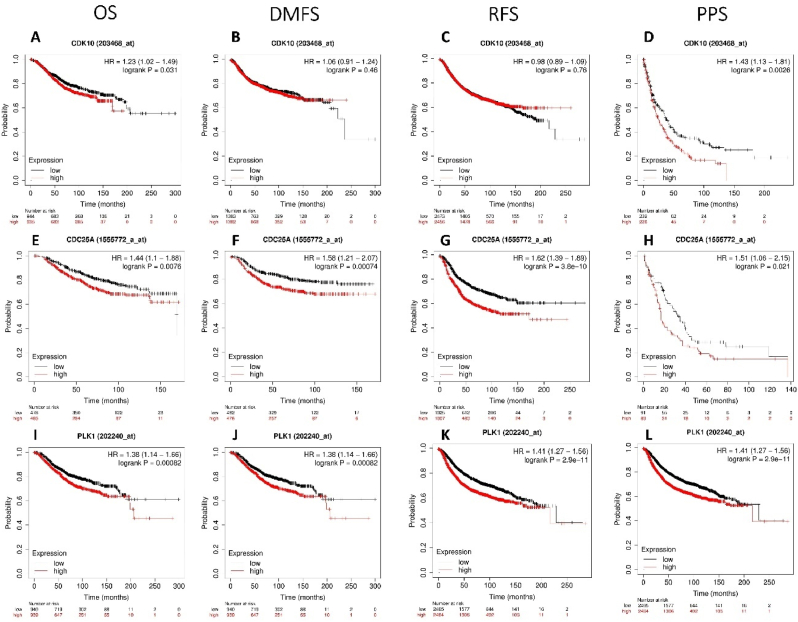
Prognostic values of CDK1, CDC25A, and PLK1 expression in breast cancer (BRCA) patients. (a) Overall survival (OS), (b) Distinct metastasis free survival (DMFS), and (c) Relapse free survival (RFS) (D) post-progression survival (PPS) in BRCA patients with high (red) and low (black) expression levels of CDK1, CDC25A, and PLK1 plotted using Kaplan–Meier plotter database at a threshold of P < 0.05.

We then analysed the prognostic value of CDK1 (203468_at), CDC25A (1555772_at), PLK1 (202240_at) in the gene chip data of Kaplan–Meier plotter. These results indicated that a lower level of CDK1 (203468_at) significantly correlated with OS (HR = 1.23 (1.02–1.49), P = 0.031) (Fig. 5A), CDC25A (203468_at) significantly correlated with DMFS (HR = 1.06 (0.91–1.24), P = 0.46) (Fig. 5B), CDK1 (203468_at) significantly correlated with RFS (HR = 0.98 (0.89–1.09), P = 0.76) (Fig. 5C), CDK1 (203468_at) significantly correlated with PPS (HR = 1.43 (1.13–1.81), P = 0.0026) (Fig. 5D), lower level of CDC25A (1555772_at) significantly correlated with OS (HR = 1.44 (1.1–1.88), P = 0.0076) (Fig. 5E), CDC25A (203468_at) significantly correlated with DMFS (HR = 1.58 (1.21–2.07), P = 0.00074) (Fig. 5F), CDC25A (1555772_at) significantly correlated with RFS (HR = 1.62 (1.39–1.89), P = 3.8e^−10^) (Fig. 5G), CDC25A (1555772_at) significantly correlated with PPS (HR = 1.51 (1.06–2.15), P = 0.021) (Fig. 5H), lower level of PLK1 (202240_at) significantly correlated with OS (HR = 1.38 (1.14–1.66), P = 0.00082) (Fig. 5I), PLK1 (202240_at) significantly correlated with DMFS (HR = 1.38 (1.14–1.66), P = 0.00082) (Fig. 5J), PLK1 (202240_at) significantly correlated with RFS (HR = 1.41 (1.27–1.56), P = 2.9e^−11^) (Fig. 5K), PLK1 (202240_at) significantly correlated with PPS (HR = 1.41 (1.27–1.56), P = 2.9e^−11^) (Fig. 5L) [51,52].

### 3.8 KEGG pathways involved in the targeted genes

**Fig. 6:**
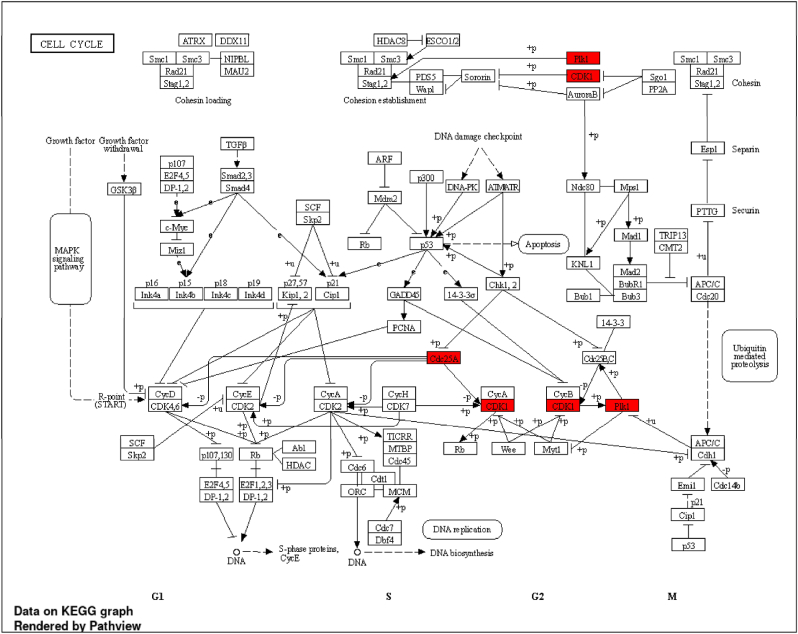
Cell cycle pathway highlighted with red colour with the targeted genes.

The pictorial representation of majorly involved genes in the results of pathways involved in case of Breast cancer is elucidated in the pathways images below. The targeted main KEGG pathway involved was found to be of cell cycle. The *in silico* and *in-vitro* cell line study in this research paper revels that CU has significantly high anticancer activity as compared to Anastrozole. Novel mechanism of action can be established based on bioinformatics, in silico and in vitro work i.e. be like *C. urens* phytoconstituent Episesamin, targets the genes like CDK1, CDC25A, PLK1 which are majorly involved in cell cycle pathway and by the downregulating those genes, the cell cycle arrest may occur in metastasis and tumour growth of breast cancer cells (Fig. 6) [53].

## 4 Discussion

In the bioinformatics, the disease genes (Breast cancer) were collected form the Gene Card database, GeneCards is a valuable repository of information regarding human genes. It serves as a comprehensive platform, consolidating information from more than 150 sources into easily understandable summaries. This extensive collection of information encompasses a wide range of topics, including gene function, protein products, linked disorders, and pertinent research. Gene Cards is also integrated with other databases, providing the opportunity to investigate the connections between genes, diseases, and biological processes [54]. Gene Cards is a potent bioinformatics tool that is highly beneficial for academics due to its emphasis on human genes and its intuitive user interface [54,55]. Breast cancer is linked to 15,474 genes from the Gene Expression Omnibus (GEO) database, a bioinformatics resource containing microarray and RNA sequencing data. Researchers utilized the MCF-7 cell line to analyse gene expression profiles (GSE226339) of three cancer stem cells (CSC) and three control groups. HR+/HER2-breast cancer's progression is influenced by the tumour microenvironment (TME), which promotes cancer stem cell enrichment. This study revealed novel roles of STAT3 in suppressing CSC enrichment and modulating immune/inflammatory responses, while p65 may compensate for STAT3's absence. Using LIMMA, 779 differentially expressed genes (DEGs) related to breast cancer stem cells were identified, highlighting the roles of STAT3 and p65 in tumorigenesis and requiring further exploration [56,57].

A total of 90 phytoconstituents of *C. urens* were gathered from published literature and online databases and subsequently screened using the SWISS ADME tool for drug-likeness evaluation. Additionally, 60 phytoconstituents and 604 associated genes were identified using SwissTargetPrediction, a bioinformatics tool that predicts protein targets based on molecular similarity to known compounds. A Venn diagram was constructed to compare differentially expressed genes (DEGs) in breast cancer with *C. urens*-targeted genes, revealing 52 common genes. These findings highlight potential molecular interactions and therapeutic targets, offering insights into the drug discovery process for breast cancer treatment [58].

The 52 identified genes were subjected to further analysis, including network construction and gene function annotation. A protein-protein interaction (PPI) network was generated to elucidate the interconnections between proteins involved in key biological processes. The PPI network provides valuable insights into the cooperative behavior of proteins within pathways and the potential impact of protein dysfunctions associated with disease progression. Such networks are essential in drug discovery, as they highlight critical proteins involved in disease mechanisms, identifying them as potential therapeutic targets. Additionally, PPI networks guide the prioritization of proteins for further functional characterization and drug development [59]. Protein-protein interaction (PPI) networks serve as powerful bioinformatics tools, providing insights into cellular machinery and supporting diverse areas of biological research. Using the STRING database, an extensive PPI network was constructed, integrating both validated and predicted interactions. STRING enables researchers to investigate biological functions and pathways, prioritize key proteins, and identify densely interconnected proteins crucial for cellular processes or diseases. The PPI network was exported to Cytoscape software, and cytoHubba identified the top 10 hub genes based on their centrality. Gene Ontology (GO) and KEGG analyses revealed potential disease networks and associated pathways. A compound-target network, alluvial plot, and compound-target pathway network (Figs. S3A–C, respectively) were also generated, linking disease pathways with *C. urens* phytoconstituents. Episesamin, a major phytoconstituent of *C. urens*, was found to target CDK1, CDC25A, and PLK1-key cell cycle regulators in breast cancer. Molecular docking using AutoDock Vina showed Episesamin exhibited the highest docking scores with these genes. CDK1, CDC25A, and PLK1 were upregulated in breast cancer compared to normal tissues, based on BRCA data analysis. This suggests Episesamin's potential to inhibit breast cancer progression by targeting these cell cycle genes [60,61].

In vitro qPCR analysis on MCF7 cells revealed significant downregulation of CDK1, CDC25A, and PLK1 genes following treatment with *C. urens* fruit extract. CDK1 plays a dual role in breast cancer, normally triggering mitosis but, when inhibited by *C. urens*, halting cell division. This inhibition can prevent uncontrolled cancer growth, though prolonged arrest may risk mitotic slippage, where unstable cells escape flawed mitosis. Targeting multiple genes, such as CDK1, CDC25A, and PLK1, effectively induces cell cycle arrest, offering a promising therapeutic strategy for breast cancer [[62], [63], [64]]. CDC25A is crucial for cell cycle progression, but its overactivity in breast cancer drives uncontrolled growth by over-activating CDKs. *C. urens* targets CDC25A, preventing it from deactivating CDK brakes, leading to cell cycle arrest. PLK1, a kinase essential for spindle formation and chromosome separation, is often overactivated in breast cancer, promoting tumour growth. Targeting PLK1 disrupts spindle assembly, causing mitotic arrest and cancer cell death. Both CDC25A and PLK1 serve as potential therapeutic targets and biomarkers, offering promise for personalized breast cancer treatments by halting uncontrolled cell growth [[65], [66], [67], [68], [69], [70]].

ROC plots were constructed to evaluate gene activity in healthy and malignant cells, identifying gene signatures that accurately distinguish between cancerous and normal tissues. A high area under the ROC curve (AUC) indicates a strong ability to identify true cancer cases, reducing false positives. ROC plots help determine optimal gene expression cutoff values for cancer diagnosis. Kaplan-Meier (KM) plotter was then used for survival analysis, assessing gene behaviour in cancer and its correlation with patient survival. KM plotter, with data from over 35,000 cancer patients, provides survival curves and statistical validation, offering insights into gene impact on survival outcomes [33,71,72]. Based on various bioinformatics, in silico docking and in vitro MCF7 cell line study reveals that the *C. urens* fruit extract targets the genes of cell cycle i.e. CDK1,CDC25A, and PLK1 in breast cancer. Thus, by down regulation of these genes inhibits the tumour cells metastasis and this establishes a novel mechanism to target the breast cancer [73].

### 4.1 Novel findings and implications for breast cancer therapy

This study reveals the therapeutic potential of *C. urens* fruit extract in breast cancer treatment. Key findings indicate that Episesamin targets and downregulates critical cell cycle genes CDK1, CDC25A, and PLK1, potentially inducing cell cycle arrest and inhibiting tumour growth. This multi-targeted approach offers promise for effective treatments, particularly for aggressive breast cancer subtypes, highlighting *C. urens* as a source of novel anticancer agents that could lead to targeted therapies with fewer side effects than conventional chemotherapy [67,74].

### 4.2 Addressing potential limitations

Despite promising findings, this study's limitations include reliance on in silico predictions, such as molecular docking and bioinformatics analysis, which cannot fully represent biological complexity. Further in vivo validation is necessary to assess the efficacy and safety of Episesamin, along with studies on potential off-target effects and pharmacokinetics. Future research should prioritize comprehensive in vivo studies and clinical trials to evaluate Episesamin's therapeutic potential and safety profile [75,76].

### 4.3 Expanding comparison with existing literature

This study enhances breast cancer research by revealing Episesamin's ability to specifically target and downregulate key cell cycle genes CDK1, CDC25A, and PLK1. This novel therapeutic strategy may offer a more robust effect against drug resistance. The findings support recent trends in targeting multiple pathways to improve treatment outcomes and underscore the potential of natural compounds in cancer therapy [77,78].

## 5 Conclusion

This study utilized a comprehensive bioinformatics approach to explore the anti-cancer mechanisms of *C. urens* fruit, focusing on the phytoconstituent Episesamin. We identified 15,474 breast cancer-associated genes, emphasizing the HR+/HER2-subtype and the role of the tumor microenvironment in metastasis via STAT3 and p65 regulation. By screening over 90 phytoconstituents, we pinpointed 60 key compounds, revealing 52 common genes targeted by both cancer mechanisms and *C. urens* constituents. In vitro experiments demonstrated that *C. urens* effectively inhibits cell cycle-related genes CDK1, CDC25A, and PLK1, validated by molecular docking and qPCR. ROC and survival analyses identified these genes as potential diagnostic and therapeutic targets. The identification of Episesamin as a potent inhibitor suggests its development as a targeted therapy for breast cancer. Future research should focus on in vivo studies, detailed molecular mechanisms, and clinical trials to assess efficacy, safety, and optimal dosing, potentially enhancing treatment outcomes and overcoming drug resistance.

## Author Contributions

GP and JC conceived the idea of writing a research and have planned the steps of research and designed manuscript structure. GP, CA and SK developed the manuscript draft which was verified and reviewed by GP and AS at the end and finial editing. All the authors have read and approved the final manuscript.

## Declaration of generative AI in scientific writing

No AI tool has been used for scientific writing.

## Funding sources

None.

## Declaration of competing interest

We declare no conflicts of interest regarding the publication of this review article. All authors have no financial or personal relationships that could influence the work.
