# Supplementary material for: Targeting cell cycle arrest in breast cancer by phytochemicals from Caryto urens L. fruit ethyl acetate fraction: in silico and in vitro validation
Source: J Ayurveda Integr Med. 2025 Mar 12;16(2):101095. doi: 10.1016/j.jaim.2024.101095 (PMC11932863; doi:10.1016/j.jaim.2024.101095)
Supplement: Multimedia component 1 [file mmc1.docx]

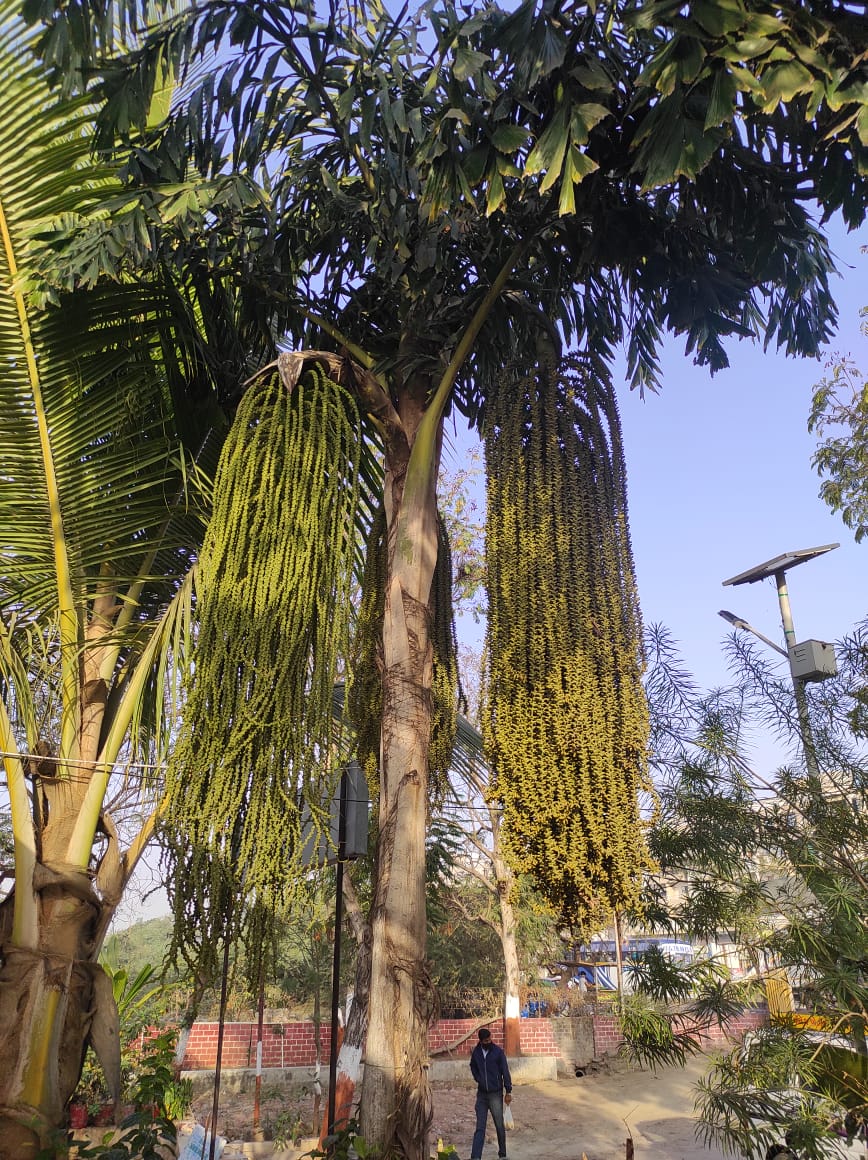


**Figure S1**: *Caryota urens* L. (Shivjata) plant and fruit image.


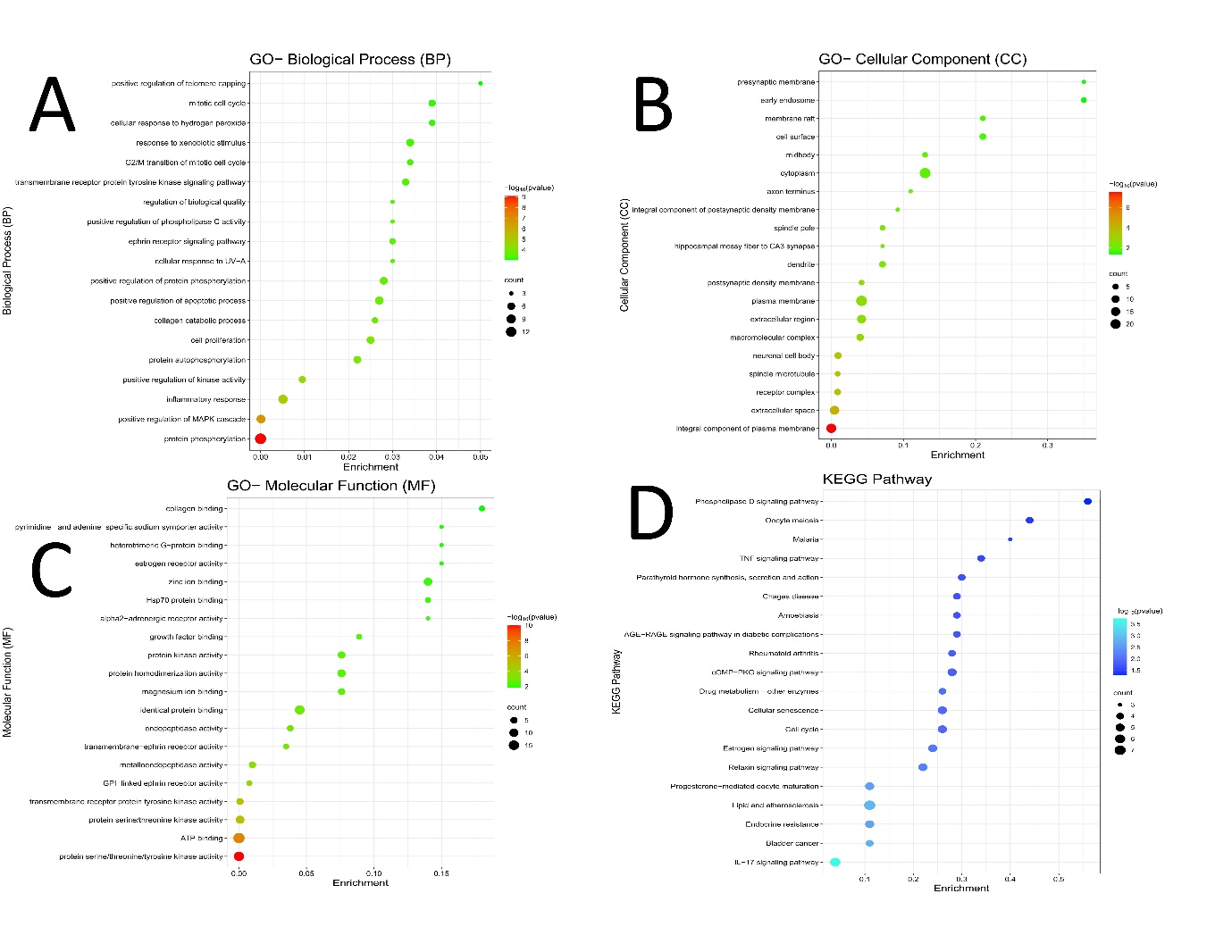


**Figure S2.** Bubble map of GO and KEGG pathway analysis (A) Biological process of GO analysis; (B) cellular components of GO analysis; (C) Molecular function of GO analysis (D) KEGG pathway analysis.


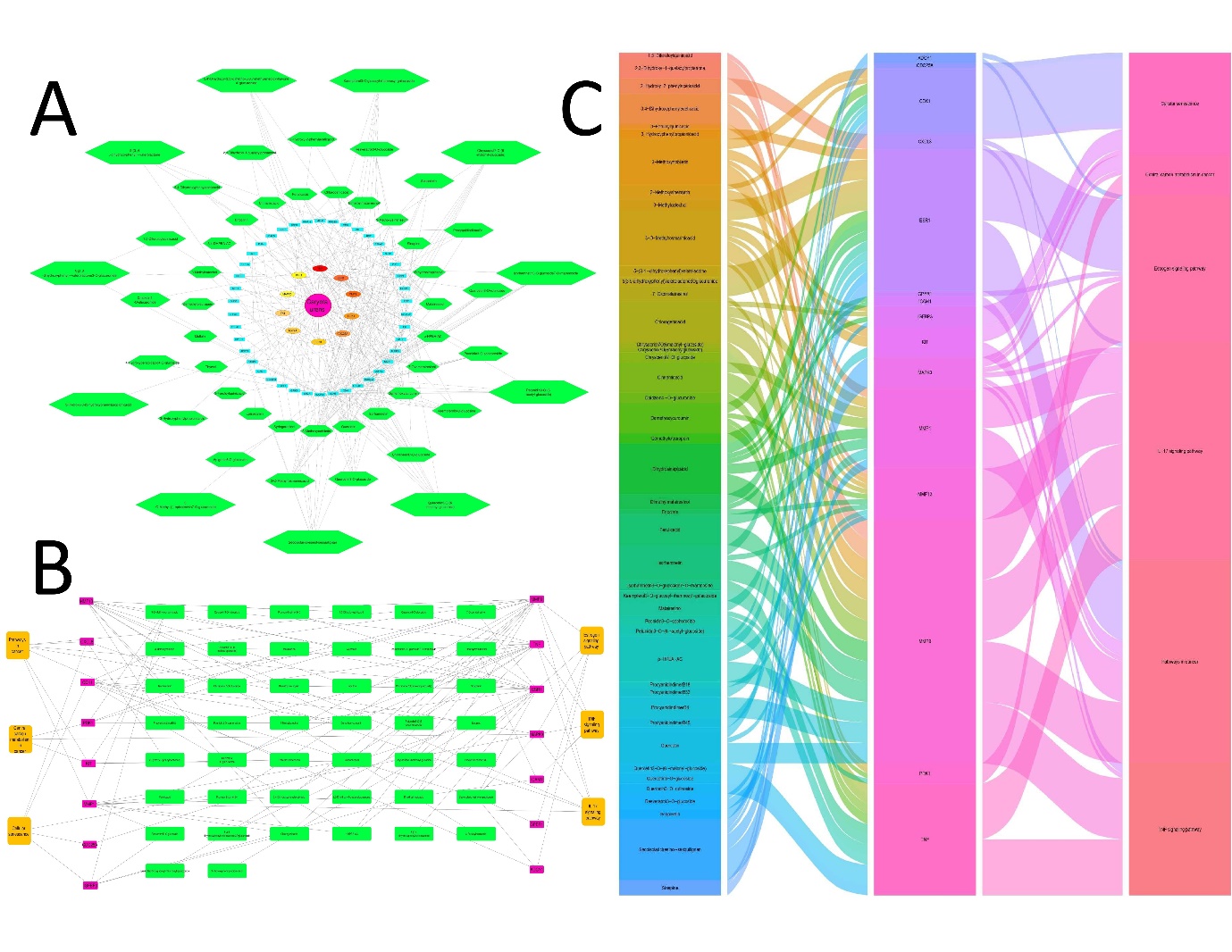


**Figure S3 : (A)** Compound-target network between active compounds and common targets of disease and compounds (green colour shows active ingredients and blue colour shows potential target genes). **(B)** Target-Pathway-Compound network between active compounds, target gen enrichment pathway. **(C)** Alluvial Plot of phytoconstituents of *C. urens with* disease (DFU) with its interacting genes.
